# The role of iliocapsularis in hip pathology: a scoping review

**DOI:** 10.1093/jhps/hnab057

**Published:** 2021-09-01

**Authors:** Masayoshi Saito, Zakir H Khokher, Yuichi Kuroda, Vikas Khanduja

**Affiliations:** Young Adult Hip Service, Department of Trauma and Orthopaedic Surgery, Addenbrooke’s - Cambridge University Hospitals NHS Foundation Trust, Hills Road, Cambridge CB2 0QQ, UK; Young Adult Hip Service, Department of Trauma and Orthopaedic Surgery, Addenbrooke’s - Cambridge University Hospitals NHS Foundation Trust, Hills Road, Cambridge CB2 0QQ, UK; Young Adult Hip Service, Department of Trauma and Orthopaedic Surgery, Addenbrooke’s - Cambridge University Hospitals NHS Foundation Trust, Hills Road, Cambridge CB2 0QQ, UK; Young Adult Hip Service, Department of Trauma and Orthopaedic Surgery, Addenbrooke’s - Cambridge University Hospitals NHS Foundation Trust, Hills Road, Cambridge CB2 0QQ, UK

## Abstract

The iliocapsularis is a relatively unheard-of muscle, located deep in the hip covering the anteromedial capsule of the hip joint. Little is known about this constant muscle despite its clinical relevance. The aims of this scoping review are to collate the various research studies reporting on the detailed anatomy and function of iliocapsularis and to demonstrate how inter-individual differences in iliocapsularis can be used as a clinical adjunct in guiding diagnosis and treatment of certain hip joint pathologies. A computer-assisted literature search was conducted based on Preferred Reporting Items for Systematic Reviews and Meta-Analyses guidelines. Our review found 13 studies including 384 cases meeting our inclusion criteria. About 53.8% of the studies involved human cadavers. The current scoping review indicates the relevant anatomy of the iliocapsularis, being a small muscle which arises from the inferior border of the anterior inferior iliac spine and anteromedial capsule of the hip joint, inserting distal to the lesser trochanter. Therefore, based upon these anatomical attachments, iliocapsularis acts as a dynamic stabilizer by tightening the anterior capsule of the hip joint. Implications of this association may be that the muscle is hypertrophied in dysplastic or unstable hips. Determining the size of the iliocapsularis could be of conceivable use in patients with hip symptoms featuring signs of both borderline hip dysplasia and subtle cam-type deformities. Although future research is warranted, this study will aid physicians to understand the clinical importance of the iliocapsularis.

## INTRODUCTION

The iliocapsularis muscle, also referred to as the iliacus minor, iliotrochantericus, iliocapsulo trochanteric or iliacus brevis, is a lesser-known but constant muscle located in the anterior aspect of the hip joint. Despite its constant presence, many clinicians are unaware of its anatomy [1]. The muscle lies deep to the rectus femoris and partially covers the anteromedial hip capsule. The first reference to the iliocapsularis appeared in 1843 in the second edition of Cruveilhier’s French anatomy text. The author originally identified this structure as an inconsistent autonomous muscle and referred to it as iliocapsulo trochanteric due to its attachments to the hip capsule and proximal to the lesser trochanter [2].

Ward *et al*. investigated the anatomy of the iliocapsularis in significant detail, as well as emphasizing its essential role in stabilization of the capsule of the hip joint. In addition, the paper highlighted its application as an important anatomical landmark intra-operatively, being a notable structure which must be identified and elevated during a Bernese periacetabular osteotomy [1]. The anterolateral border of the iliocapsularis has been described as the ideal location for capsulotomy in anterior total hip arthroplasty, and its anteromedial border can be used during a modified Smith-Peterson approach to identify the joint capsule in periacetabular osteotomy [3]. Furthermore, when performing an iliopsoas tenotomy, the iliocapsularis is useful in identifying the iliopsoas tendon during anterolateral or direct lateral approach [4]. The iliocapsularis muscle is also relevant whilst performing a T-capsulotomy during hip arthroscopy, as the desired site for incision is between the fibres of the iliocapsularis and the gluteus minimus to prevent iatrogenic injury to the medial and lateral femoral circumflex arteries [5, 6]. Although the iliocapsularis has been used as a key landmark in multiple surgical procedures including periacetabular osteotomy, anterior hip arthroplasty and hip arthroscopy, there is a paucity in the literature focusing on detailed anatomy, function and clinical relevance of this muscle.

To establish a more complete understanding of the lesser-known iliocapsularis, a scoping review was planned to map the literature on the topic, identifying sources of evidence, key topics and gaps in research. Scoping reviews are beneficial in providing a broad, extensive review of literature on a topic and is advantageous to a systematic review when conducting a comprehensive examination of a topic rather than exploring a specific question [7]. Current and future plausible clinical applications of the muscle have also been documentated and are of great interest to the hip surgeon. The specific aims of this scoping review, therefore, were to collate the various research studies reporting on the anatomy of the iliocapsularis and how inter-individual differences may be used to guide clinical practice bearing in mind the functional relevance of the muscle.

## MATERIALS AND METHODS

The methodology for this scoping review was based on the framework outlined by Arksey and O’Malley [8] and ensuing recommendations made by Levac *et al* [7, 9]. The review included the following five key phases: (i) identifying the research question; (ii) identifying relevant studies; (iii) study selection; (iv) charting the data and (v) collating, summarizing and reporting the results. The optional ‘consultation exercise’ of the framework was not conducted.

### Research question

This review was guided by the primary question, ‘What has been currently described in the literature regarding the iliocapsularis and its relevance to hip pathology?’ The secondary questions were set as follows:

- What is the detailed anatomy of the iliocapsularis i.e. length, width, depth (thickness), location (proximal and distal attachment), and blood and nerve supply?
- What are the functions of the muscle?
- How may inter-individual differences be used to guide clinical practice, bearing in mind the anatomy and function of the muscle?

### Data sources and search strategy

Two reviewers (M.S. and Z.H.K.) searched the online databases [Pubmed (Medline), EMBASE and Cochrane Library] for literature describing the iliocapsularis. The Preferred Reporting Items for Systematic Reviews and Meta-Analyses (PRISMA) guidelines were used for designing this study [10]. Database search was conducted on 1 October 2019 and retrieved articles from database inception to the search date. The individual study eligibility criteria were established *a priori.* We used medical subject headings including the following search terms: iliocapsularis, iliacus minor, iliotrochantericus, iliocapsulo trochanteric and iliacus brevis. Terms were connected by the Boolean operator ‘OR’ (Appendix A). Results were pooled, and duplicate searches were excluded by having two reviewers (M.S. and Z.H.K.) independently review the titles and abstracts. The remaining search results were divided equally between two reviewers (M.S. and Z.H.K.) and reviewed in duplicate applying the inclusion and exclusion criteria. Any discrepancies at the full-text stage were resolved by consensus between the two reviewers. If a consensus could not be reached, a third more senior reviewer (V.K.) was consulted to resolve the discrepancy.

### Study screening

The inclusion and exclusion criteria are shown in Table I. Both reviewers independently abstracted the relevant study data from the final pool of included articles and recorded these data on a spreadsheet designed *a priori*.

**Table I.** Inclusion and exclusion criteria applied to articles identified in the literature

Inclusion criteria
(i) All levels of evidence
(ii) Written in the English language
(iii) Studies on humans
(iv) Studies published in a peer-reviewed journal
Exclusion criteria
(i) Reviews, systematic reviews
(ii) Technical notes
(iii) Abstract-only studies
(iv) Book chapters

### Assessment of level of evidence

The level of evidence was evaluated based on the guidelines by the Oxford Centre for Evidence-Based Medicine [11]. Levels I, II, III, IV and V evidence were elibigible for inclusion in the scoping review.

### Date characterization

A proforma was developed by the authors to extract study characteristics such as publication year, the level of evidence, the number of hips, distribution of right and left hips, mean age with range (years), gender distribution, Body Mass Index (BMI) and specific comments. This form was reviewed by the research team and pretested by all reviewers (M.S., Z.H.K., Y.K. and V.K.) before implementation, resulting in minor modifications to the proforma. The characteristics of each full-text article were extracted by two independent reviewers (M.S. and Z.H.K.).

### Data summary and synthesis

The data were compiled in a single spreadsheet and imported into Microsoft Excel 2013 (Microsoft Corporation, Redmond, WA) for validation and coding. Statistical analysis in this study focused on descriptive statistics to summarize the data. Frequencies and percentages were utilized to describe nominal data.

## RESULTS

### Search and selection of studies

**Fig. 1.**
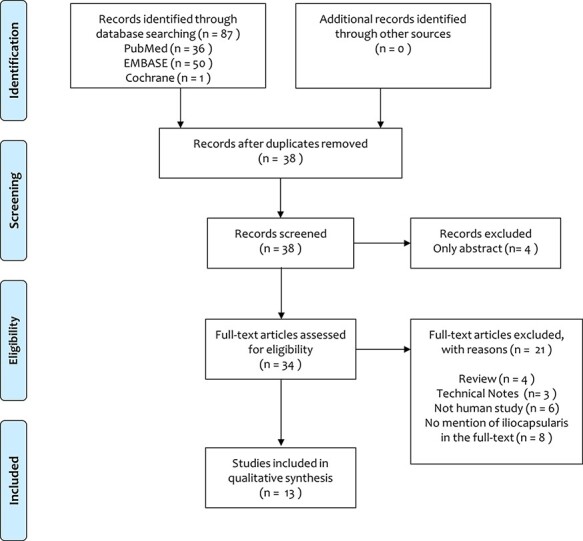
The PRISMA flow diagram.

Flowchart of the literature search using PRISMA guidlines is shown in Fig. 1. The initial search of the online databases resulted in 87 total studies. A systematic screening and assessment of eligibility identified 13 full-text articles including 384 cases that satisfied the inclusion and exclusion criteria.

### General characteristics of included studies

The oldest study included in this review was published in 1950, and approximately 75% of the included studies (10 of 13 studies) were published in or after 2014. The level of evidence in all studies was III, IV or V, and 53.8% of studies (7 of 13 studies) were human cadaveric studies. Details of the 13 studies included are shown in Table II. The studies included in this scoping review are broadly divided into Anatomy, Function and Clinical Relevance, which are described in detail.

**Table II.** Study characteristics

	*Author*	*Y* *ear*	*LOE*	*Objectives, number of hip*		*M* *ean age*	*G* *ender (% male)*	*BMI*	*Right hip (%)*	*C* *omments*
1	Das	1950	5	1 Cadaver		−	−	−	0	Iliocapsularis is a rare muscle in the human body and when present it represents a detached part of the iliacus muscle
2	Ward	2000	4	20 Fresh cadavers	No prior hip surgery	−	25	−	−	Iliocapsularis originates in part from the inferior border of the AIIS, but the main origin arises from an elongated attachment to the anteromedial hip capsule and inserts just distal to the lesser trochanter
3	Babst	2011	4	45 Hips with pain	Dysplasia	34 ± 9.7 (17–49)	45	25 ± 5 (18–37)	47	Increased thickness, width, circumference, CSA and partial volume of the iliocapsularis, and less fatty infiltration in the patients with dysplasia compared with excessive acetabular coverage
				40 Hips with pain	Pincer FAI	33 ± 11.0 (17–49)	31	23 ± 4 (18–32)	45	
4	Philippon	2014	4	14 Fresh cadavers	No prior hip surgery, degenerative change and dysplasia	58 (47–65)	86	24.6 (19.2–32.1)	57	The iliocapsularis originated from the inferior facet of the AIIS. The inferolateral corner of the footprint of the iliocapsularis origin was located 12.5 mm (95% CI, 10.1–15.0 mm) from the acetabular rim
5	Walters	2014	4	11 Fresh cadavers	No prior hip surgery	72.3 (67–95)	−	24.6 (14.5–36.2)	−	The iliocapsularis had the most significant capsular contributions and was adherent to the entire length of the anteromedial capsule beginning at its origin at the inferior aspect of the AIIS to its insertion just distal to the lesser trochanter
6	Haefeli	2015	3	45 Hips with pain	Dysplasia	34 ± 10 (17–49)	45	25 ± 5 (18–37)	47	The iliocapsularis-to-rectus-femoris ratio for CSA, thickness, width and circumference were increased in hips with radiographic evidence of dysplasia (ratios ranging from 1.31 to 1.35) compared with pincer FAI (ratios ranging from 0.71 to 0.90; *P* < 0.001) and compared with the control group
				40 Hips with pain	Pincer FAI	33 ± 11.0 (17–49)	31	23 ± 4 (18–32)	45	
				30 Asymptomatic hip	control	54 ± 12 (29–75)	50	26 ± 8 (14–37)	66	
7	Cooper	2015	4	11 Fresh cadavers		79.2 (67–95)	−	24.6 (14.5–36.2)	−	Iliocapsularis had large direct capsular attachments; dimensions defined as being 73.8 mm in length and 16.1 mm in width
8	Wyatt	2016	3	18 Hips with pain	Stable dysplasia	32 ± 13 (14–55)	39	−	−	Iliocapsularis volume did not discriminate between treatment groups (periacetabular osteotomy or FAI surgery) with radiographic evidence of LCEA of 25° or less. However, a larger iliocapsularis volume was associated with greater antetorsion
				21 Hips with pain	Unstable dysplasia	31 ± 10 (15–46)				
				20 Asymptomatic hips	Age-matched controls	37 ± 11 (15–52)				
9	Lawrenson	2017	4	15 Asymptomatic hips	No prior hip surgery	22 ± 2	67	−	−	The greatest muscle activity, which is the highest of electromyographic amplitude, by intramuscular electrode insertion occurred during isometric hip flexion at 90° and the lowest activity during hip extension at 0°
10	Ricci	2019	5	1 Hip with pain		30	100	−	0	Synovial bursitis between the rectus femoris direct tendon and iliocapsularis was likely the cause of anterior hip pain in this case
11	Lawrenson	2019	4	14 Asymptomatic hips		22.4 ± 1.8	71	23.6 ± 3.4	−	Iliocapsularis demonstrates a consistent burst of muscle activity around toe-off in natural walking, with inconsistent muscle activity observed in mid-late stance. In shortened strides, the burst of muscle activity in mid to late stance became more consistent and had increased amplitude
12	Elvan	2019	4	21 Formalin-fixed foetuses		29 ±3.9 week (25–36)	43	−	−	Iliocapsularis is a constant muscle also in the foetal period. Its dimensions, location and course over the anteromedial part of the hip joint capsule suggest its prominent support to hip joint stability
13	Tsutsumi	2019	4	17 Fresh cadavers	No prior hip surgery	81	56	−	−	The origin of the iliocapsularis corresponded with the shallow groove at the anteromedial surface of the AIIS, which was identified by micro-CT

LOE, level of evidence; CT, computed tomography; values are expressed as mean ± standard deviation and range in parentheses.

### Anatomy

Das and Singh in 1950 described the iliocapsularis as an exceptionally rare muscle in the human body [12]. However, a recent anatomical study involving human formalin-fixed foetuses recorded a prevalence of this muscle at 92% in the foetal period. Elvan *et al*. found that the iliocapsularis was consistently located lateral to the iliopsoas, deep to the rectus femoris, medial to the gluteus minimus, as well as overlying the anteromedial part of the capsule of the hip joint [13]. As such, it has now been recognized as an individual and constant muscle. The following anatomical features of the iliocapsularis have been reported in detail in the literature.

#### Proximal attachment of the iliocapsularis

Elvan and colleagues included 21 formalin-fixed foetuses in their study [13], noting three different variations of the proximal attachment of iliocapsularis:

- Below the proximal attachment of the rectus femoris muscle found in 54% of cases;
- Forming a common tendon with rectus femoris on the anterior inferior iliac spine (AIIS) found in 26% of cases;
- Forming an arch along the superior–medial–inferior sides of the proximal attachment of the rectus femoris muscle, found in the remaining 20% of cases.

**Fig. 2.**
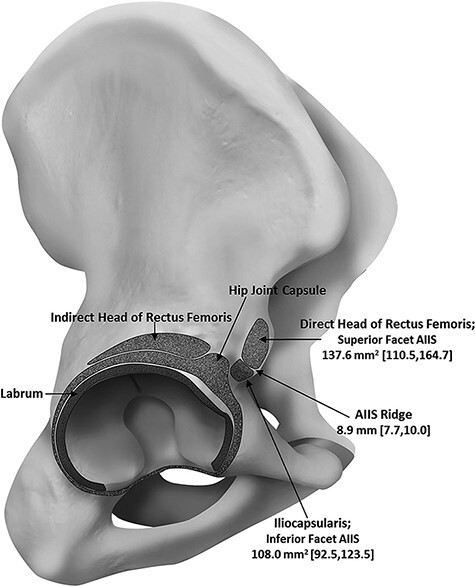
An illustration indicating the various anatomical attachments of the iliocapsularis, direct and indirect head of rectus femoris, labrum and capsule of the hip joint to the right hip. Mean measurements of the area of the superior and inferior facets of the AIIS, plus the mean width of the AIIS ridge are indicated with 95% confidence intervals shown in brackets. Illustration retrieved from Phillipon *et al*. (2014) [14].

**Fig. 3.**
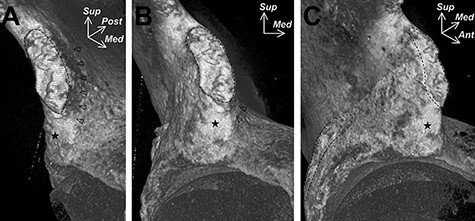
An image derived from micro-computed tomography indicating the shallow groove of the anteromedial surface of the AIIS (indicated by use of arrowheads) where the iliocapsularis was suggested to arise from. The dashed lines correspond roughly to the superior portion of the AIIS and anterolateral wall of the ilium. The star indicates the smooth impression at the inferior portion of the AIIS. Ant= anterior, Med= Medial, Post = posterior, Sup = superior. Image describes the anteromedial (A), anterior (B) and anterolateral (C) aspects of the right hip. Image retrieved from Tsutsumi *et al*. (2019) [16].

Muscle fibres originating from the anteromedial part of the capsule of the hip joint were also recognized to be constant in all specimens. However, in adult studies, the proximal attachment of the iliocapsularis still remains a controversial issue. Ward *et al*. described the iliocapsularis as originating from the anteromedial hip capsule as well as the inferior border of the AIIS [1]. Substantiating this, Philippon *et al*. described the division of the AIIS into superior and inferior facets, divided by a horizontal osseous ridge (AIIS ridge) [14]. The inferior facet occupied 44% of the total area of the AIIS, and Philippon *et al*. affirmed the iliocapsularis to originate from the inferior facet of the AIIS in all 14 of their adult cadaveric specimens (Fig. 2) [14]. Similarly, Walters *et al*. supported the origin to be the inferior aspect of the AIIS in a cadaveric study [15]. On the contrary, a recent cadaveric study showed that the origin of the iliocapsularis corresponded with the shallow groove at the anteromedial surface of the AIIS, which was identified by micro-computed tomography (Fig. 3) [16].

#### Muscle belly

Iliocapsularis muscle length measured between 12 and 13 cm in 20 fresh adult cadavers [1]. Its length was identified to be longer in females than males during the foetal period (*P* = 0.031) and acknowledged to be wider on the right side (*P* = 0.029) [13].

Measurements of iliocapsularis width, depth (thickness), circumference and cross-sectional area (CSA) are described in Table III.

**Table III.** Measurements of iliocapsularis width, depth (thickness), circumference and CSA in included studies

*Subjects*	*21 Formalin-fixed foetuses*	*20 Cadaveric specimens*	*45 Hips with dysplasia*	*40 Hips with pincer FAI*	*P Value (dysplasia versus pincer FAI)*	*45 Hips with dyspsia*	*40 Hips with pincer FAI*	*P Value (dysplasia versus pincer FAI)*	*30 healthy control*	*45 Hips with dysplasia*	*40 Hips with pincer FAI*
*Measurement* *location level*	*its mid-length*	*4 cm below the AIIS*				*T* *he first section inferior to the femoral head*			*F* *emoral head centre*		
Muscle width, cm	1.9 ± 0.4 (1.3–3.3)	1.8–2.5	2.7 ± 0.6 (1.7–4.0)	2.0 ± 0.5 (0.9–3.9)	<0.001	2.6 ± 0.5 (1.7–3.7)	2.2 ± 0.5 (1.1–3.1)	<0.001	2.1 ± 0.5 (1.2–3.3)	2.3 ± 0.6 (1.4–3.4)*^,^^§^	1.9 ± 0.3 (0.9–2.4)^§^
Muscle depth (thickness), cm	No date	0.4–1.0	1.6 ± 0.4 (1.0–2.8)	1.4 ± 0.4 (0.8–2.4)	0.01	2.1 ± 0.4 (1.2–2.9)	1.7 ± 0.4 (1.0–2.7)	<0.001	1.4 ± 0.4 (0.7–2.1)	1.7 ± 0.5 (0.7–2.7)*^,^^§^	1.3 ± 0.2 (0.8–2.3)
Circumference, cm	No date	No date	7.2 ± 1.3 (5.1–11.5)	5.5 ± 1.1 (2.7–7.5)	<0.001	7.7 ± 1.3 (5.6–11.4)	6.2 ± 1.2 (4.0–8.2)	<0.001	5.9 ± 1.2 (3.1–8.0)	6.9 ± 1.8 (3.3–11.3)*^,^^§^	6.0 ± 0.9 (3.9–7.9)^§^
CSA, cm^2^	No date	No date	2.5 ± 0.9 (1.2–5.2)	1.8 ± 0.6 (0.6–3.0)	<0.001	3.1 ± 1.0 (1.7–5.9)	2.3 ± 0.9 (0.9–4.4)	<0.001	1.9 ± 0.8 (0.6–4.3)	2.1 ± 1.0 (0.6–2.7)*	1.5 ± 0.5 (0.4–2.2)^§^

values are expressed as mean ± standard deviation and range in parentheses.

* significant difference compared with the pincer group (*P* < 0.05);

§ significant difference compared with the control group (*P* < 0.05).

#### Distal attachment of iliocapsularis

Contrary to the proximal attachment, the insertion appears to be non-controversial, with its location found consistently 1.5 cm distal to the lesser trochanter in the femur [1]. Walters *et al*. corroborated this finding, describing its insertion just distal to the lesser trochanter [15]. Furthermore, during the foetal period the insertion of the iliocapsularis was consistently found to be distal to the lesser trochanter in all specimens [13].

#### Relationship with capsule of the hip joint

The attachment between the iliocapsularis and the anteromedial hip capsule appeared to be robust; only through sharp dissection could these muscle fibres be separated from this tight association. The iliocapsularis had the largest contribution along the anteromedial capsule, with the dimensions of capsular contributions being 73.8 ± 27.3 mm × 16.1 ± 4.4 mm [1, 17].

#### Blood supply

Blood supply to the iliocapsularis muscle is via two arteries. One is the profunda femoris artery via small branches, and the other is the lateral femoral circumflex artery via small branches emanating from the hip capsule [1].

#### Nerve supply

Innervation of the iliopcasularis is achieved from the femoral nerve, by way of a small nerve branch arising as the femoral nerve crosses the superficial surface of the iliocapsularis following piercing of the iliacus [13].

### Function

**Fig. 4.**
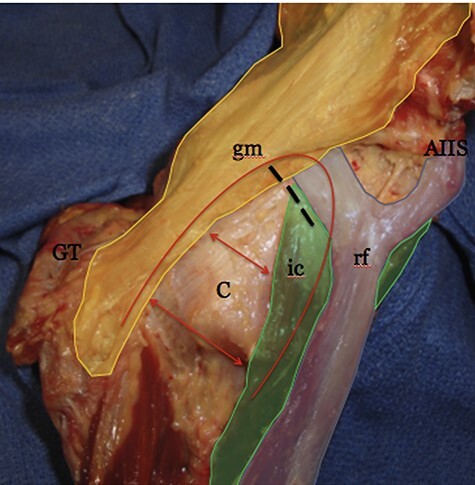
An image demonstrating the borders of the stability arc, outlined in red. The borders are composed of the dynamic muscular stabilisers [iliocapsularis (ic), gluteus minimus (gm) and reflected head of the rectus femoris (rf)] and static stabilizing limbs of the iliofemoral ligament (ILFL-h and ILFL-v) which lie directly beneath the gluteus minimus and iliocapsularis, respectively. The greater trochanter (GT), AIIS, and anterior capsule (C) are labelled for orientation purposes. The red arrows demonstrate the tension which arises during dynamic movements across the anterior capsule as the limbs of the arc contract to stabilize the hip joint. The black dashes indicate the location of a standard interportal medial capsulotomy. Image retrieved from Walters *et al*. (2014) [15].

Because of the orientation, substantial pericapsular attachment and stretching over the anteromedial capsule [1, 15, 17, 18], the iliocapsularis has been suggested to actively stabilize the anterior hip. In addition, the dimensions, location and course over the capsule of the hip joint of this muscle support the theory that it tightens the anterior hip capsule and stabilizes the femoral head in the foetal period [13]. As three muscles have direct attachments to the anterior capsule, including iliocapsularis, anterior gluteus minimus and rectus femoris, the iliofemoral ligament is thought to work in conjunction with these muscles to form a ‘stability arc’ to constrain anterior translation of the femoral head (Fig. 4) [15].

Based on the first electromyography study involving the iliocapsularis, the greatest muscle activity occurred during hip flexion, particularly with the hip flexed to 90°, supporting the hypothesized role of iliocapsularis in control of anterior femoral head translation and retaining the femoral head in a centripetal orientation during the position of hip flexion [19]. In an additional electromyography study [20], iliocapsularis demonstrated a consistent burst of muscle activity during toe-off in natural walking, with inconsistent muscle activity observed in the mid-late stance. In shortened strides, the burst of muscle activity in mid-late stance became more consistent and with increased amplitude. This suggested a potential role for iliocapsularis in augmenting capsular tension to aid hip flexion, hinging on the presumption that initiation of hip flexion in gait is thought to occur by stored energy from anterior passive structures rather than flexor muscle activity [21].

## DISCUSSION

This is the first scoping review elucidating the anatomy, function and clinical relevance of the iliocapsularis; 13 studies were identified following a clear inclusion and exclusion criteria using PRISMA guidelines. The majority of the included studies examined anatomy, and these detailed anatomical findings have implications for the understanding of the functional role of the iliocapsularis. Based upon these anatomical attachments, the most important finding of this review was that iliocapsularis acts as a dynamic stabilizer by tightening the anterior capsule of the hip joint. Implications of this association may be that the muscle is hypertrophied in dysplastic or unstable hips.

### Clinical relevance

Determining the size of the iliocapsularis could be of conceivable use in patients with hip symptoms featuring signs of both borderline hip dysplasia and subtle cam-type deformities. In the last 10 years, hip arthroscopy has grown to become an integral part of the treatment of hip pathology [22]; however, arthroscopic surgery for hip dysplasia remains controversial [23]. It can be often difficult for the clinician to define the predominant pathophysiological issue in symptomatic hips with features of both borderline dysplasia [defined as a lateral centre-edge angle (LCEA) between 20° and 25°] [24, 25] and cam-type femoroacetabular impingement (FAI) [22, 26]. Several other factors suggestive of hip dysplasia, such as hypertrophy of the labrum, presence of ganglia in the labrum, decentration of the femoral head, decreased head sphericity and epiphyseal index, plus an increased epiphyseal angle can aid in the decision making process as to whether the symptoms are due to dysplasia or impingement [27, 28]. Furthermore, Babst *et al*. suggested that pre-operative evaluation of the morphological features of the iliocapsularis can also be used as an adjunct for decision-making when treating symptomatic patients with borderline dysplasia or FAI [18]. Muscular hypertrophy and a diminished degree of fatty infiltration of iliocapsularis e.g. can be indicative of hip dysplasia rather than FAI in borderline cases where the underlying symptomatic pathology is unclear. This is due to the presumed function of iliocapsularis in stabilization of the femoral head based upon its attachments. Thus, in the deficient acetabulum, iliocapsularis would be speculated to be hypertrophied [18]. In addition, Haefeli *et al*. evaluated not only absolute measurements including muscle width, depth (thickness), circumference and CSA, but also evaluated the diagnostic value of the relative size of the iliocapsularis muscle in relation to the rectus femoris muscle to distinguish between dysplasia and pincer-type FAI [29]. The authors suggested the iliocapsularis-to-rectus-femoris ratio may be a valuable secondary sign of dysplasia. This parameter can also be used as an adjunct for clinical decision-making in cases with borderline hip dysplasia and an associated small cam-type deformity where the underlying pathomechanism is unclear. A raised iliocapsularis-to-rectus-femoris ratio would be suggestive of dysplasia and a superior indicator of dysplasia in comparison to solely measuring iliocapsularis volume due to elimination of confounding factors such as gender and size.

Wyatt *et al*. demonstrated that iliocapsularis volume (in a cohort of patients with LCEA of 25° or less) was indifferent between two symptomatic groups, who were either recognized to have unstable hips or clinically apprehended as possessing stable hips and FAI acknowledged to be the leading pathology [30]. Those with medically diagnosed unstable hips underwent periacetabular osteotomy [31], whilst an open or arthroscopic surgical approach for FAI was indicated for those with stable hips. Despite this, a larger iliocapsularis volume was favourably associated with greater femoral antetorsion [30]. The reason for this likely attributable to patient selection, with comparisons being drawn between unstable and stable borderline hips; the anatomical difference being only minimal in this study [30].

There are certain aspects of the iliocapsularis which are still not fully understood and require further work. Firstly, there have been no studies which look at a direct comparison between consequential hypertrophy of lliocapsularis and clinical outcome following surgery of the hip. If hypertrophy of lliocapsularis is proven as a poor prognostic factor pre-operatively in patients having arthroscopic surgery, then hypertrophy picked on MRI can be an adjunct to recommending acetabular osteotomy rather than arthroscopic surgery. Secondly, besides the iliocapsularis-to-rectus-femoris ratio shown in this review as an assessment of hip instability, recent studies reported that the Femoro-Epiphyseal Acetabular Roof Index or labral length may be a poor prognostic factor for following hip arthroscopy [24, 32, 33]. These findings often indicate multiple lesions. However, the prognostic value of these findings with regard to inferior outcome following hip arthroscopy is poorly understood. Certainly further comparative studies are necessary to define independent factors confirming hip instability and prognostic factors leading to inferior outcomes following hip arthroscopy. Lastly, Domb *et al*. asserted that hypertrophy of the iliocapsularis may be the cause of the unique 3 o’clock anterior labral injury observed in his study [34]. The distinct position of this lesion is suggested to be unrelated to FAI or dysplasia due to its focal position; these conditions usually result in lesions in the anterosuperior region of the labrum. Hyperactivity or hypertrophy of iliocapsularis may be providing a shearing force resulting in a repetitive traction injury on this focal region of the anterior capsulo-labral complex [34]. This suggests that hip dysplasia and the consequential hypertrophy of iliocapsularis may result in further pathological circumstances outside of the direct repercussions of dysplasia.

### Limitations

There are several limitations to this study. Most of our reviewed studies were case series by design, which limited the average level of evidence. Given the limited data, a breakdown by sex could not be provided. Thus, we could not conclude if there are any differences between genders. In addition, the asymptomatic participants in our reviewed studies may already have sufficient stability provided by passive structures, limiting the necessity for iliocapsularis to actively contribute to hip joint stability. To evaluate whether iliocapsularis activity supplements joint stability in pathology, further investigation in populations with hip dysplasia or FAI would be indicated. We would recommend further research to examine this. Furthermore, the search criteria were limited to Embase, PubMed (Medline) and Cochrane Library databases’ studies that were published in English. Therefore, there may be a possible selection bias in the current study.

## CONCLUSION

The current scoping review describes the relevant anatomy, possible roles, as well as the clinical relevance of the iliocapsularis muscle. Although there are multiple theories encompassing the origin of the iliocapsularis, the consensus is that the inferior border of the AIIS is where the muscle originates from. The iliocapsularis has the most significant contribution to the anteromedial capsule, and the insertion appears to lie just distal to the lesser trochanter. The muscle acts as a dynamic stabilizer by tightening the anterior capsule of the hip joint, implying that it may be hypertrophied in dysplastic or unstable hips. Determining the size of the iliocapsularis may be of use in symptomatic patients with dual hip pathology, who possess features of both borderline hip dysplasia and subtle cam-type deformities. Although future research is warranted, this study will help physicians to understand the clinical importance of the iliocapsularis.

## APPENDIX A.

### SEARCH STRATEGY

**Table:**

*PubMed: 35 Studies*		*EMBASE: 50 Studies*		*Cochrane: 1 Study*	
Strategy	Studies	Strategy	Studies	Strategy	Studies
iliocapsularis OR iliacus minor OR iliotrochantericus OR iliocapsulo trochanteric OR iliacus brevis	36	1. iliocapsularis.mp	21	1. iliocapsularis	1
		2. iliacus minor.mp	17	2. iliacus minor	0
		3. iliotrochantericus.mp	5	3. iliotrochantericus	0
		4. iliocapsulo trochanteric.mp	0	4. iliocapsulo trochanteric	0
		5. iliacus brevis.mp	8	5. iliacus brevis	0
		6. 1 or 2 or 3 or 4 or 5	50	6. 1 or 2 or 3 or 4 or 5	1
